# Compliance to colchicine treatment and disease activity in Familial Mediterranean Fever (FMF) patients in Middle/Black Sea Region of Turkey (in Çorum region)

**DOI:** 10.1186/1546-0096-13-S1-P81

**Published:** 2015-09-28

**Authors:** Y Karaaslan, Į Dogan, A Omma, S Can Sandikci

**Affiliations:** 1 Hitit University Medical Faculty, Rheumatology, Çorum, Turkey; 2 Ankara Numune Education and Research Hospital, Rheumatology, Ankara, Turkey; 3 Çorum Education and Research Hospital, Rheumatology, Çorum, Turkey

## Background and question

Colchicine is the gold standard treatment for prevention of inflammatory attacks and prevention of reactive amyloidosis in FMF. However, noncompliance to colchicine treatment is common among FMF patients. On the other hand, the disease may not be controlled in some patients despite use of full dose colchicine. In this study, we aimed to investigate the rates of disease control, compliance to colchicine treatment, and need for an additional treatment despite use of full dose colchicine in patients with FMF in Çorum region where FMF is common in Turkey.

## Methods

The patients with FMF who admitted to Rheumatology Clinic established 1 year ago in a tertiary medical center located in Corum province, and followed up with the diagnosis of FMF for at least 6 months were analyzed. A total of 96 consecutive patients who admitted to our center in last 3 months, fulfilled Tel-Hashomer FMF diagnostic criteria, and on colchicine treatment were included in the study.

## Results

The mean age of the patients was 33.1±10.8 years, female/male ratio was 65/31, and the mean disease duration was 20.6±14.4 years. 39.6% of the patients included in the study had at least three or more attack in last 12 months (2.2% of the patients had 25 or more attack, 4.2% patients had 12-24 attack, 18.8% patients had 5-11 attack and 11.5% patients had 3-4 attack in last 12 months).

The mean attack frequency in last 12 months was determined 3.2. The levels of acute phase reactants were high in 27.3% of the patients examined in attack free period. 4.2% patients had markedly proteinuria.

Use of colchicine was very regular (>90%) in 38.5%, predominantly regular (75-90%) in 26%, and moderately regular (50-74%) in 21.9% of the patients while 8.3% of the patients reported that they used colchicine sometimes, or only during attacks (90%) use of colchicine, 10.8% of the patients reported at least 3 or more attack in last 12 months, and 16.2% of the patients had high acute phase reactant levels in attack free period. 1 patients were on anti-IL-1 antagonist treatment.

## Conclusions

In real life, the disease was not under control in 39.6% of FMF patients who used colchicine. 35.5% of FMF patients did not use the drug regularly, and an additional treatment was needed in 10.8% of the patients despite regular use of the agent in a region of Turkey where FMF is common (in Çorum region).

